# The E-Wave Deceleration Rate E/DT Outperforms the Tissue Doppler-Derived Index E/e' in Characterizing Lung Remodeling in Heart Failure with Preserved Ejection Fraction

**DOI:** 10.1371/journal.pone.0082077

**Published:** 2013-12-03

**Authors:** T. Dung Nguyen, Yasushige Shingu, Michael Schwarzer, Andrea Schrepper, Torsten Doenst

**Affiliations:** 1 Department of Cardiothoracic Surgery, Jena University Hospital – Friedrich Schiller University Jena, Jena, Germany; 2 Department of Cardiovascular Surgery, Hokkaido University, School of Medicine, Sapporo, Japan; Virginia Commonwealth University, United States of America

## Abstract

**Background:**

Diastolic dysfunction in heart failure with preserved ejection fraction (HFpEF) may result in pulmonary congestion and lung remodeling. We evaluated the usefulness of major diastolic echocardiographic parameters and of the deceleration rate of early transmitral diastolic velocity (E/DT) in predicting lung remodeling in a rat model of HFpEF.

**Methods and Results:**

Rats underwent aortic banding (AoB) to induce pressure overload (PO). Left ventricular hypertrophy fully developed 2 weeks after AoB. At 4 and 6 weeks, the lung weight-to-body weight ratio (LW/BW), a sensitive marker for pulmonary congestion and remodeling, dramatically increased despite preserved fractional shortening, indicating the presence of HFpEF. The time course of LW/BW was well reflected by E/DT, by the ratio of early to late transmitral diastolic velocity (E/A) and the deceleration time of E (DT), but not by the ratio of transmitral to mitral annular early diastolic velocity (E/e'). In agreement, the best correlation with LW/BW was found for E/DT (r = 0.76; p<0.0001), followed by E/A (r = 0.69; p<0.0001), DT (r = −0.62; p<0.0001) and finally E/e' (r = 0.51; p<0.001). Furthermore, analysis of receiver-operating characteristic curves for the prediction of increased LW/BW revealed excellent area under the curve values for E/DT (AUC = 0.98) and DT (AUC = 0.95), which are significantly higher than that of E/e' (AUC = 0.82). In a second approach, we also found that the new parameter E/DT correlated well with right ventricular weight index and echocardiographic measures of right ventricular systolic function.

**Conclusions:**

The novel parameter E/DT outperforms the tissue Doppler index E/e' in detecting and monitoring lung remodeling induced by pressure overload. The results may provide a handy tool to point towards secondary lung disease in HFpEF and warrant further clinical investigations.

## Introduction

Based on recent epidemiological studies, up to half of all heart failure patients present an ejection fraction (EF) greater than 40% [1], [2]. With its high prevalence, growing incidence and poor prognosis, heart failure with preserved ejection fraction (HFpEF) has become a significant public health problem [3], [4]. Several cardiac impairments may contribute to the pathophysiology of HFpEF. These include left ventricular (LV) diastolic dysfunction, abnormal ventricular-arterial coupling, chronotropic incompetence, etc. [5].

Chronic backward failure in HFpEF causes pulmonary congestion and consequently lung remodeling. In experimental models of HFpEF, this is characterized by increased lung weight in the absence of pulmonary water retention [6]. Because LV diastolic dysfunction is mostly involved in HFpEF [7], we expected that evaluating diastolic function might help predict pathological lung remodeling in HFpEF.

Diastolic function can be assessed non-invasively by echocardiography using Doppler and tissue Doppler techniques. While the application of diastolic parameters has been suggested to diagnose heart failure-induced lung disease in the latest guidelines [8], [9], their usefulness in this context has not yet been validated. Furthermore, their diagnostic reliability in general is a matter of debate. For instance, although the ratio of transmitral to mitral annular early diastolic velocity (E/e') has been suggested as a non-invasive surrogate of LV filling pressure [10], recent studies found only a weak or even no correlation between E/e' and LV diastolic pressure [11], [12].

In the present study, we evaluated for the first time the diagnostic value of main echocardiographic diastolic parameters in predicting lung remodeling using a well established rat model of HFpEF. Furthermore, we aimed to test our hypothesis that the deceleration rate of early transmitral diastolic velocity (E/DT) can be used to assess heart failure-induced lung remodeling (see Fig. 9 and Appendix S1 for the rationale of this hypothesis).

## Methods

### Animals

Male Sprague-Dawley rats were obtained from Charles River (Germany) and fed a standard laboratory chow and water ad libitum at 21°C with a light cycle of 12 h.

### Ethics statements

All animals received humane care in compliance with the “Principles of Laboratory Animal Care” formulated by the National Society for Medical Research and the “Guide for the Care and Use of Laboratory Animals” prepared by the Institute of Laboratory Animal Resources and published by the National Institutes of Health (NIH Publication No. 86-23, revised 1996). The experimental protocols were approved by the local Institutional Animal Care and Use Committee (Institut für Versuchstierkunde und Tierschutz, Friedrich-Schiller-Universität Jena).

### Experimental design

Rats were subjected to chronic pressure overload by aortic arch banding (AoB). At 2, 4 and 6 weeks after AoB, we conducted echocardiography and determined the heart weight-to-body weight ratio (HW/BW) and the lung weight-to-body weight ratio (LW/BW) as ex vivo markers for cardiac hypertrophy and heart failure-induced lung remodeling, respectively, in AoB and their age-matched control groups. The LW/BW index was used due to its high sensitivity despite simple measurability [6]. Echo measures obtained at 4 and 6 weeks were correlated with their corresponding LW/BW values and used to generate receiver operating characteristic curves (ROC curves). In a separate group of rats with pressure overload, serial echocardiography was performed at 2, 4 and 6 weeks to additionally characterize the time course of the assessed echocardiographic measures in a longitudinal manner (follow-up group).

### Surgical procedures

Our surgical protocol for the induction of hypertrophy and heart failure in rats has been described in detail earlier [13]. Briefly, 3-week-old rats were anaesthetized and ventilated with room air during the operation. Following a partial sternotomy and removal of the thymus, a titanium clip was placed around the aortic arch between the brachiocephalic trunk and the left carotid artery. The clip had a remaining opening of 0.35 mm, causing progressively increased afterload. After the procedure, animals were extubated and kept on warming blankets until full consciousness was regained.

### Echocardiographic image acquisition

**Figure 1:**
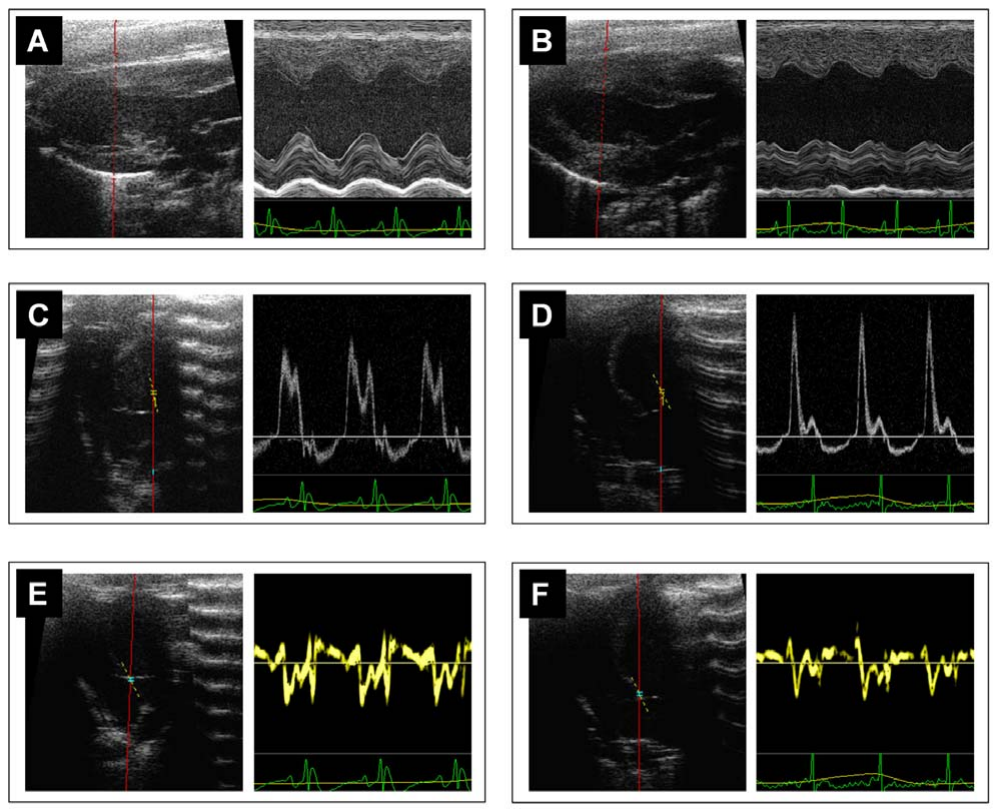
Representative echocardiographic images of control and pressure overloaded rats at 6 weeks after aortic banding. B-mode and M-mode assessments of LV morphology and systolic function in control (**A**) and pressure overload rats (**B**). Four chamber view and transmitral Doppler pattern in control (**C**) and pressure overloaded rats (**D**). Four chamber view and tissue Doppler spectrum in control (**E**) and pressure overloaded rats (**F**).

Rats were anesthetized by intramuscular injection of a combination of midazolam hydrochloride, medetomidin hydrochloride and fentanyl (2, 0.15, 0.005 mg/kg). Subsequently, the left anterior chest was shaved and rats were placed in a supine position on an examination pad with embedded ECG leads allowing for simultaneous monitoring of ECG, heart rate and respiration throughout an imaging session. Echocardiography was performed using a Vevo 770 rodent ultrasound system (VisualSonics, Canada), equipped with high-resolution mechanical transducers. We used a 25 MHz-scanhead (RMV710B) for small rats (2 weeks after AoB) and a 17 MHz-scanhead (RMV716) for larger rats (4 and 6 weeks after AoB). Left ventricular size and systolic function were assessed using 2D-guided M-mode from the parasternal long-axis according to the recommendations of the American Society of Echocardiography [14]. The M-mode cursor was carefully placed perpendicularly to the long axis of the left ventricle at the level where the greatest LV end diastolic dimension could be seen. Mitral inflow pattern was assessed from the apical 4-chamber view using pulsed-wave Doppler with a sample volume of 1 mm placed at the mitral leaflet tips. Similarly, tissue Doppler assessments were conducted from the apical 4-chamber view with the sample volume placed at the septal insertion site of the mitral leaflet. The lateral mitral annulus was not assessed due to unreliable visibility. The ultrasound system has a separate tissue Doppler mode with default settings optimized for tissue Doppler imaging so that only gain adjustments were necessary. Due to the angle dependence of Doppler techniques, various insonation angles were tested to ensure that the maximal velocities were obtained. To cover the longitudinal excursion of the mitral annulus, further Doppler angle corrections of 20°–40° were required. According to the manufacturer, high accuracy can be achieved with angle correction fewer than 60°. See Fig. 1 for examples of Doppler and tissue Doppler imaging. Right ventricular (RV) systolic function was assessed by characterizing the longitudinal systolic motion of the tricuspid lateral annulus from the apical 4-chamber view [15]. For each assessment mode, 3 to 6 images were obtained and stored in digital format for later analysis. All examinations were completed within 20 minutes. At the end of each imaging session, rats were immediately sacrificed for necropsy or anesthesia was stopped (follow-up group) by subcutaneous injection of atipamezole, flumazenil and naloxone (1, 0.1, 0.03 mg/kg).

### Necropsy

For the correlation of echocardiographic parameters with LW/BW (at 4 and 6 weeks), rats were euthanized immediately after the echocardiogram. The heart and the lung were carefully isolated from surrounding tissues, washed in saline and weighed. Subsequently, the lung was minced and dried in an incubator at 50°C for 24 hours to determine dry weight. The whole heart and the ventricles were separately weighed after the remaining blood was removed. In addition, the postmortal position of the clip was inspected to verify the induction of pressure overload.

### Histological assessments

Masson's trichrome stain was conducted to visualize collagen fibers using a commercially available kit (Sigma-Aldrich) according to the manufacturer's protocol. This approach was sensitive enough to detect LV myocardial fibrosis induced by pressure overload (Fig. S1). Lung tissue density, interstitial and periarteriolar collagen content was quantified using the program ImageJ. To measure lung tissue density, representative overview images of lung sections were analyzed (Fig. S3). Threshold was adjusted to include all stained content (lung tissue and infiltrating cells). Lung tissue density was calculated as fractional area of the thresholded structures. To measure interstitial or periarteriolar relative collagen content, color threshold was set to selectively label the blue-stained connective tissue. This threshold was then applied to quantify the fractional area of interstitial or periarteriolar collagen fibers. See Fig. S4 for examples.

### Echocardiographic image analysis

Images were analyzed off-line by a single observer blinded to necropsy results using VisualSonics Cardiac Measurements (Version 17). For each parameter, measurements were performed from three to six different cardiac cycles and the values were averaged.

M-mode measurements from the parasternal long-axis view included anterior wall thickness in diastole and systole (AWd and AWs), left ventricular end-diastolic dimension (LVEDD), left ventricular end-systolic dimension (LVESD), posterior wall thickness in diastole and systole (PWd and PWs). It is noteworthy that in contrast to human echocardiogram, the interventricular septum in rats is normally not visible in the long-axis view.

Endocardial fractional shortening (eFS) was used to evaluate systolic function and was calculated by: 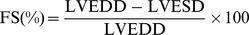



Left ventricular mass index (LVMI) was calculated from M-mode measurements and body weight (BW) using the following formula: 
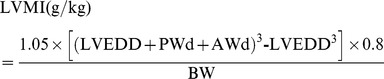



Regression analysis revealed that calculated values of LVMI correlated well with necropsy results of the hypertrophy index HW/BW (r = 0.78; p<0.0001).

Mitral inflow Doppler measurements included early (E) and late (A) diastolic peak velocities, deceleration time of E (DT) and isovolumic relaxation time (IVRT). DT was defined as the time between peak E and the intersection of the upper deceleration slope and the zero baseline. Derived parameters of mitral inflow Doppler involved the ratios E/A and E/DT.

In tissue Doppler mode, we measured the early (e′) and late (a′) diastolic peak velocities as well as the systolic (s′) peak velocity of the septal mitral annulus. ECG was applied to identify the waves. Derived parameters of tissue Doppler assessments included the ratios E/e' and e'/a'.

The functional characterization of the right ventricle involved measurements of tricuspid annular plane systolic excursion (TAPSE) and peak systolic velocity of the tricuspid lateral annulus (Ts'). We did not assess RV fractional area change due to incomplete visibility of the RV apex.

### Statistical analysis

The intra- and interobserver variability of the assessed echo parameters were calculated and presented as Bland-Altman plots using Sigma Plot 12.0. We used One Way ANOVA to compare the parameters among AoB and control groups at different time points, One Way Repeated Measures ANOVA to analyze the serial echo results of the follow-up group, and the Holm-Sidak method for pairwise multiple comparisons. Pearson correlation and ROC curve analysis were used to evaluate the diagnostic potential of the investigated echo parameters. Data are expressed as mean ±SEM. All statistical tests were performed using SigmaStat 3.5. Statistical significance was assumed if p<0.05.

## Results

**Figure 2:**
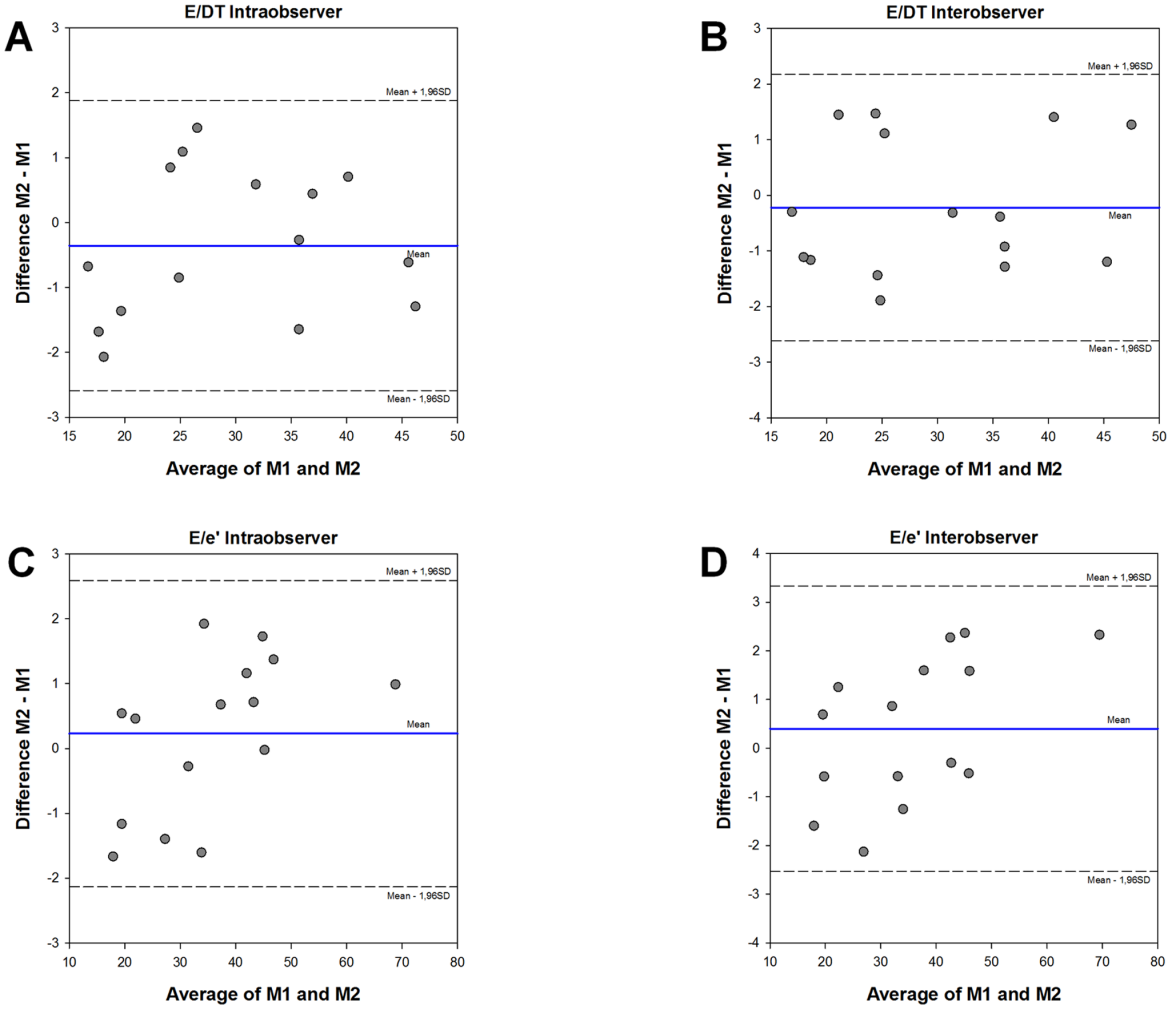
Intra- and interobserver variability of E/DT (A, B) and E/e' (C, D) presented as Bland-Altman plots. M: measurement. Dashed lines present the limits of agreement (Mean ±1.96 SD).

The intra- and interobserver variability of the assessed echo parameters were low in general (<10%). See Bland-Altman plots in Fig. 2 for the intra- and interobserver variability of E/DT and E/e'.

### Pressure overload increased LW/BW despite preserved systolic function

Table 1 and table 2 display selected necropsy and echo parameters of pressure overloaded and control rats at 2, 4 and 6 weeks. Rats with pressure overload weighed less at 2 and 4 weeks but not at 6 weeks. Heart rate was significantly elevated in pressure overload at 4 and 6 weeks, possibly due to higher activity of the sympathetic nervous system. Among Doppler and tissue Doppler measures presented in table 1, only A-wave and e′ were significantly reduced at 6 weeks.

**Table 1:** Body weight, heart weight, lung weight and heart rate of control and pressure overloaded rats at 2, 4 and 6 weeks after aortic banding.

	2 Weeks		4 Weeks		6 Weeks	
	Control (n = 5–11)	AoB (n = 8–19)	Control (n = 5–12)	AoB (n = 10–19)	Control (n = 5–7)	AoB (n = 16–19)
BW (g)	159.28±3.38	115.55±2.94*	227.27±5.14	199.00±5.29*§	291.56±4.08	271.85±6.58§
HW (mg)	636.92±27.42	667.44±26.59	835.00±20.57	1258.25±66.46*§	935.20±24.34	1851.29±52.30 *§#
LW (mg)	997.28±53.00	864.86±36.90	1239.08±29.37	1572.05±164.83§	1423.60±60.10	2997.32±179.88*§#
HR (bpm)	296.17±12.19	306.50±7.92	282.20±9.02	319.42±5.83*	257.20±11.96	292.53±3.53*#

Data are mean ±SEM.

AoB: aortic banding; BW: body weight; HW: heart weight; LW: lung weight; HR: heart rate.

Significantly different vs. age-matched control: *, vs. AoB at 2 weeks: §, vs. AoB at 4 weeks: #.

**Table 2:** Selected Doppler and tissue Doppler parameters of control and pressure overloaded rats at 2, 4 and 6 weeks after aortic banding.

	2 Weeks		4 Weeks		6 Weeks	
	Control (n = 5–11)	AoB (n = 8–19)	Control (n = 5–12)	AoB (n = 10–19)	Control (n = 5–7)	AoB (n = 16–19)
E (cm/s)	80.06±1.77	94.75±6.86	84.33±4.74	98.28±3.35	89.90±3.18	98.18±3.37
A (cm/s)	52.79±3.52	45.06±5.69	52.84±4.74	37.10±4.07	50.33±3.04	24.05±3.68*§#
IVRT (ms)	35.42±4.09	26.67±2.41	31.80±1.54	25.55±1.29	32.79±1.20	27.58±1.47
s′ (cm/s)	2.26±0.10	1.86±0.13	2.48±0.09	2.07±0.11	2.90±0.35	1.78±0.11
e′ (cm/s)	3.99±0.31	2.63±0.25	4.12±0.24	3.23±0.23	5.50±0.64	3.06±0.26*
a′ (cm/s)	2.48±0.20	2.24±0.22	2.40±0.19	2.25±0.14	2.67±0.33	2.03±0.19

Data are mean ±SEM.

AoB: aortic banding; IVRT: isovolumic relaxation time.

Significantly different vs. age-matched control: *, vs. AoB at 2 weeks: §, vs. AoB at 4 weeks: #.

**Figure 3:**
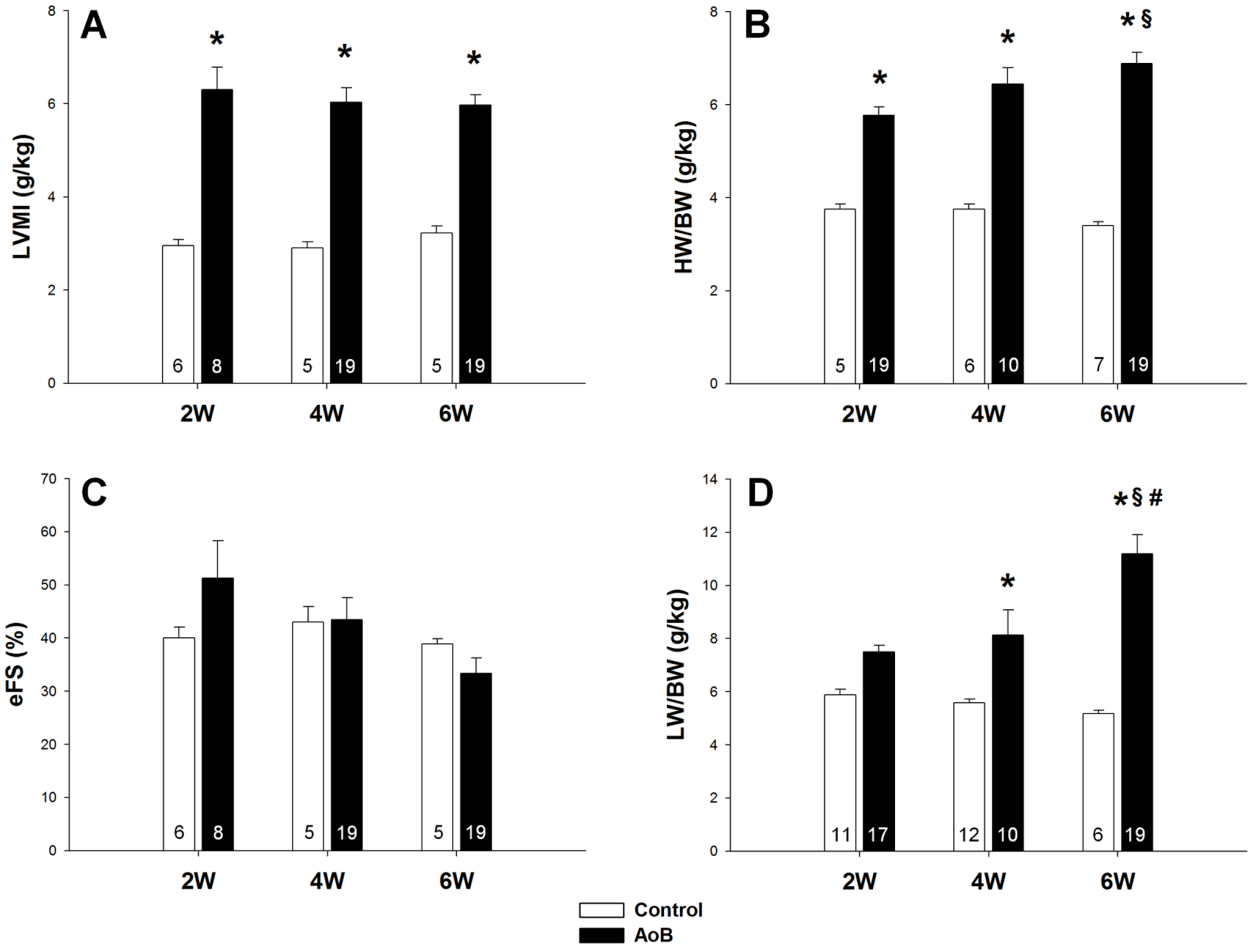
Six weeks of pressure overload induced hypertrophy and lung remodeling despite preserved systolic function. (**A**): Left ventricular mass index (LVMI), (**B**): heart weight-to-body weight ratio (HW/BW), (**C**): endocardial fractional shortening (eFS) and (**D**): lung weight-to-body weight ratio (LW/BW) of control and pressure overloaded rats at 2, 4 and 6 weeks after aortic banding (AoB). Data are mean ±SEM. Significantly different vs. age-matched control: *, vs. AoB at 2 weeks: §, vs. AoB at 4 weeks: #. The numbers in the bars indicate group size.

Figure 3A and 3B demonstrate the changes in LVMI and HW/BW of pressure overloaded rats at 2, 4 and 6 weeks after AoB. Both parameters indicate that hypertrophy fully developed as early as 2 weeks and was constantly present at 4 and 6 weeks. Systolic function as shown by eFS was unchanged at all three time points (Fig. 3C). However, the LW/BW ratio, an excellent marker for pressure overload-induced lung remodeling [6], tends to rise as early as 2 weeks and was significantly elevated at 4 and 6 weeks (Fig. 3D), indicating the presence of HFpEF. Of note, the increase in lung weight does not reflect pulmonary water retention but rather structural remodeling of the lung because its wet-to-dry ratio was unchanged by pressure overload (data not shown) [6]. Also refer to Fig. S2–S4 for macroscopic and histological presentation of lung remodeling.

### Mitral inflow parameters but not E/e' reflected the temporal changes in LW/BW

**Figure 4:**
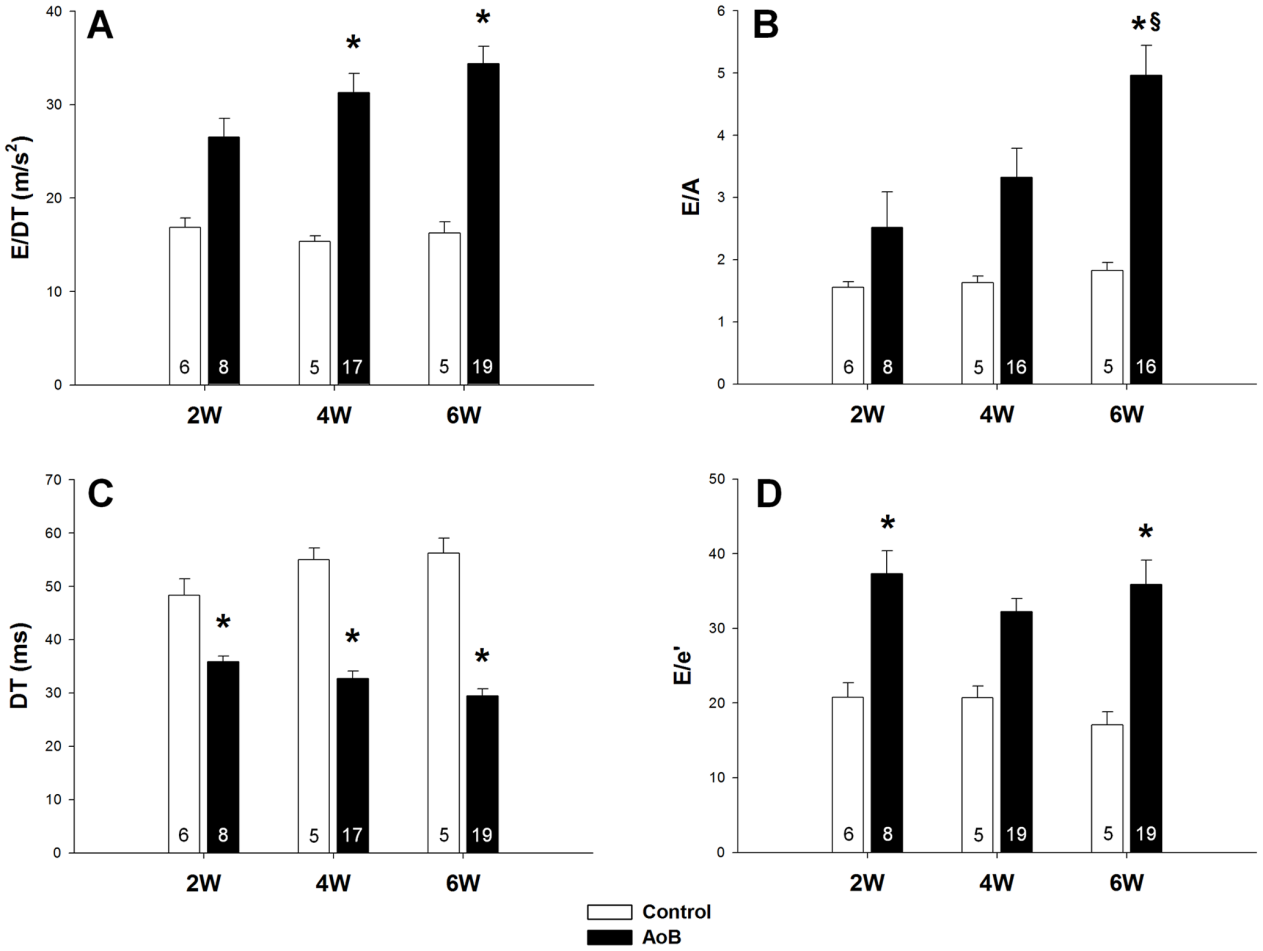
Pressure overload caused changes in transmitral and tissue Doppler parameters of diastolic function. Changes in the deceleration rate E/DT (**A**), the E/A ratio (**B**), the deceleration time DT (**C**) and the E/e' ratio (**D**) in control and pressure overloaded rats at 2, 4 and 6 weeks after aortic banding (AoB). Data are mean ±SEM. Significantly different vs. age-matched control: *, vs. AoB at 2 weeks: §. The numbers in the bars indicate group size.

Figure 4 shows the time courses of mitral inflow Doppler and tissue Doppler parameters of diastolic function. At 2 weeks after AoB, both E/DT and E/A were substantially elevated. At 4 and 6 weeks, E/DT was significantly increased and the change in E/A became significant at 6 weeks (Fig. 4A, 4B). Therefore, the temporal changes in E/DT and E/A are consistent with those of LW/BW. Although DT was significantly decreased at all time points, it does not show a clearly worsening trend as seen in LW/BW (Fig. 4C). Compared to E/DT, E/A and DT, the tissue Doppler parameter E/e' appears to reflect the changes in LW/BW worst. For instance, E/e' was significantly increased at 2 weeks but not at 4 weeks although the opposite applies to LW/BW. Furthermore, whereas LW/BW clearly indicates a worsening trend of lung remodeling from 2 to 6 weeks, E/e' values at 2 and 6 weeks were almost identical (Fig. 4D).

**Figure 5:**
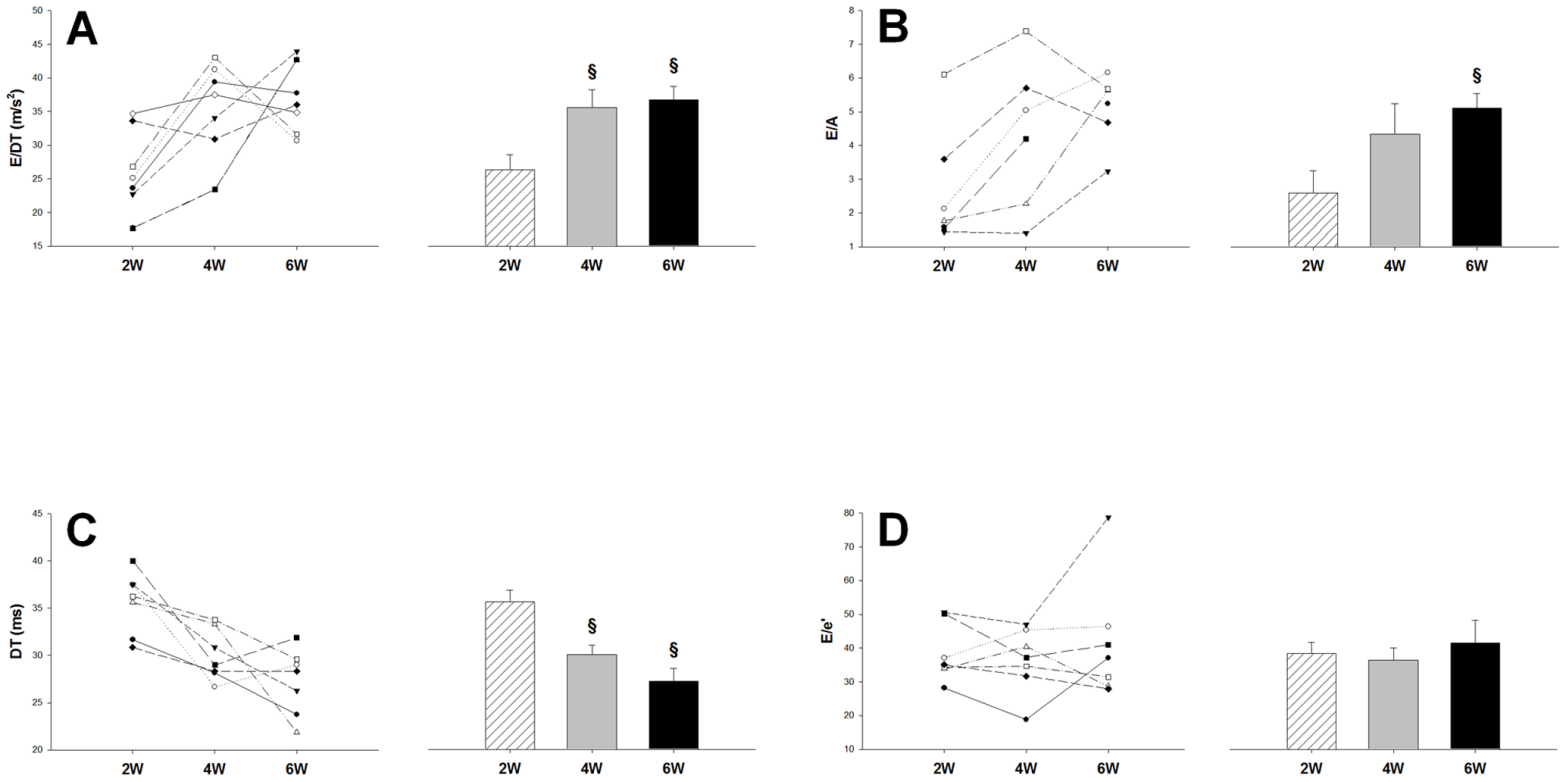
Temporal changes in transmitral and tissue Doppler parameters of diastolic function in a flow-up group with pressure overload. Time course of the deceleration rate E/DT (**A**), the E/A ratio (**B**), the deceleration time DT (**C**) and the E/e' ratio (**D**). Data are mean ±SEM. Significantly different vs. AoB at 2 weeks: §. Group size n = 7.

In a separate group of pressure overloaded rats, we performed serial echocardiography to assess the usability of these echo parameters in follow-up examination. E/DT, E/A and DT clearly described a worsening trend from 2 to 6 weeks (Fig. 5A, 5B, 5C), which is consistent with the LW/BW data (Fig. 3D). In contrast, E/e' remained unchanged at pathological values at all three time points (Fig. 5D).

### E/DT correlated best with LW/BW

**Figure 6:**
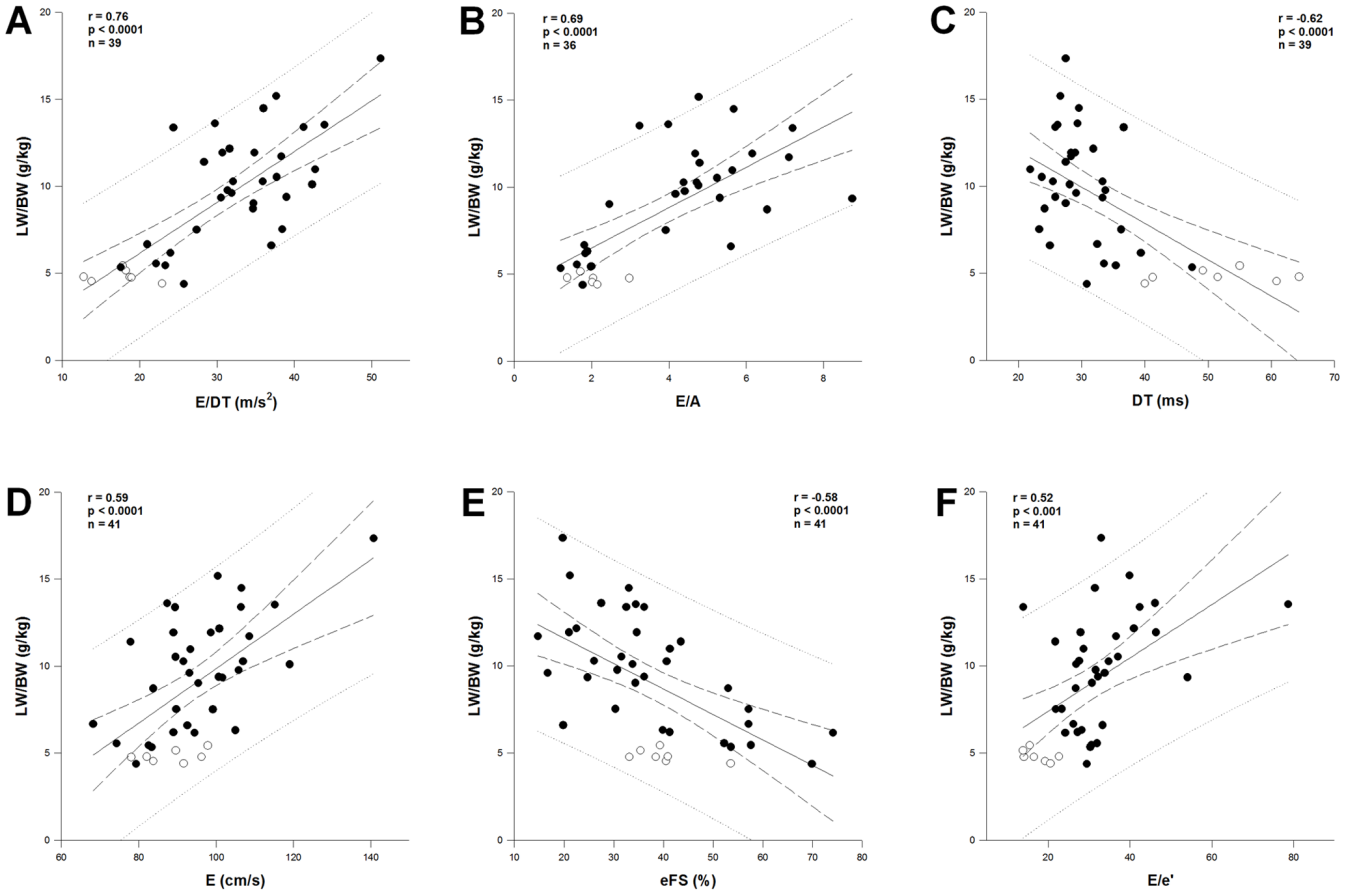
Correlation of echocardiographic parameters with the lung weight-to-body weight ratio. Pearson correlation of the deceleration rate E/DT (**A**), the E/A ratio (**B**), the deceleration time DT (**C**), peak early diastolic velocity E (**D**), endocardial fractional shortening eFS (**E**) and the E/e' index (**F**) with LW/BW. White dots: control; Black dots: aortic banding.

We used 34 pressure overloaded rats at 4 and 6 weeks and 7 control rats at 6 weeks for correlation analysis. We assessed the correlation at 4 and 6 weeks because LW/BW was most likely to increase at these two time points (Fig. 3D). A-wave was not present in 5 rats and DT could not be reliably measured in 2 rats due to fusion of E-wave and A-wave. Figure 6 displays the correlation of various echo parameters with LW/BW. The best correlation was found for E/DT with the highest correlation coefficient (r = 0.76; p<0.0001), followed by E/A (r = 0.69; p<0.0001). The correlation coefficients of the non-indexed parameters DT (r = −0.62), E (r = 0.59) and eFS (r = −0.58), all with p<0.0001, are comparable. Interestingly, E/e' showed the lowest correlation coefficient with LW/BW (r = 0.52; p<0.001).

### Mitral inflow Doppler parameters outperformed E/e' in detecting lung remodeling

To evaluate the diagnostic potential of E/DT, E/A, DT and E/e', we applied receiver operating characteristic (ROC) curves generated from the same data used for correlation analyses. Based on LW/BW values of control objects at 4 and 6 weeks (Fig. 3D), the presence of lung remodeling was assumed if LW/BW >6 mg/g.

**Figure 7:**
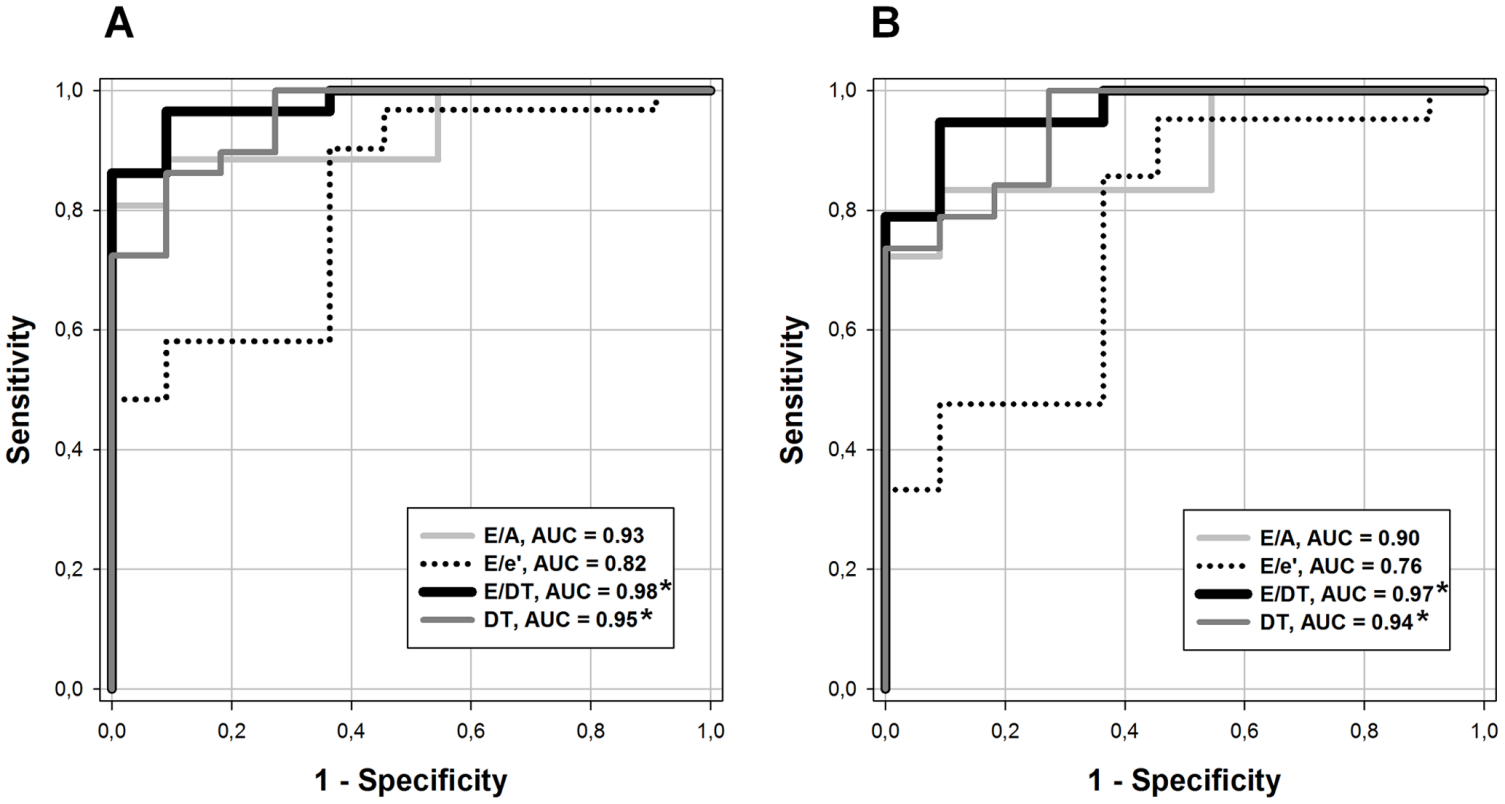
Sensitivity and specificity of transmitral and tissue Doppler parameters of diastolic function. ROC curves of E/DT, E/A, DT and E/e' for the detection of lung remodeling irrespective of eFS (**A**) or with preserved eFS (**B**). AUC: area under the curve. *: significantly different vs. E/e'.

Figure 7A presents the sensitivity and specificity of the four echo parameters for the detection of lung remodeling, irrespective of systolic function. The areas under the curve (AUC) of E/DT (0.98), DT (0.95) and E/A (0.93) reveal an excellent diagnostic accuracy of these parameters. With a cut-off value of 23.65 m/s^2^, for example, E/DT could predict lung remodeling with a sensitivity of 97% and a specificity of 91%. In contrast, the AUC of E/e' was acceptable (0.82), but significantly smaller than that of E/DT or DT, as analyzed by the Delong and Clarke-Pearson method [16].

For the detection of lung remodeling in HFpEF, defined as LW/BW >6 mg/g and eFS >30%, a similar constellation was found (Fig. 7B). The diagnostic accuracy of E/DT (AUC = 0.97) and DT (AUC = 0.94) remained outstanding and significantly superior to that of E/e' (AUC = 0.76).

### E/DT correlated well with lung tissue density and right ventricular changes

**Figure 8:**
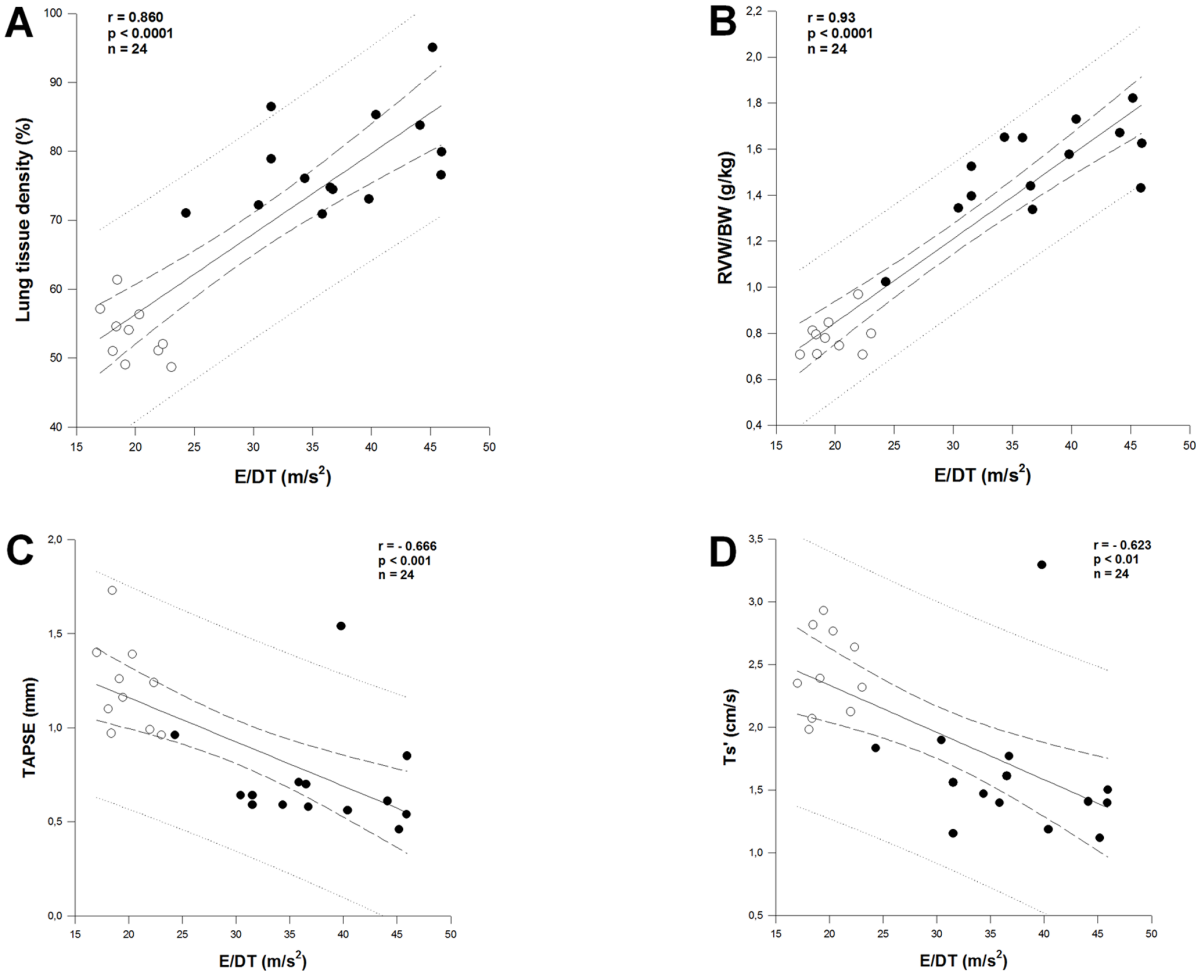
Correlation of E/DT with lung tissue density and right ventricular changes. Pearson correlation of E/DT with lung tissue density (**A**), with the right ventricular weight-to-body weight ratio (RVW/BW) (**B**), with tricuspid annular plane systolic excursion (TAPSE) (**C**), and with peak systolic velocity of the lateral tricuspid annulus (Ts') (**D**). White dots: control; Black dots: aortic banding.

To test if E/DT may predict histological characteristics of lung remodeling, we assessed lung tissue density, interstitial and periarteriolar relative collagen content. We found no changes in interstitial or periarteriolar collagen content at all three assessment time points (Table 3). However, lung tissue density was substantially increased at 2 weeks and this change became significant at 4 and 6 weeks (Table 3). Furthermore, E/DT correlated well with this histological marker of lung remodeling (Fig. 8A).

**Figure 9:**
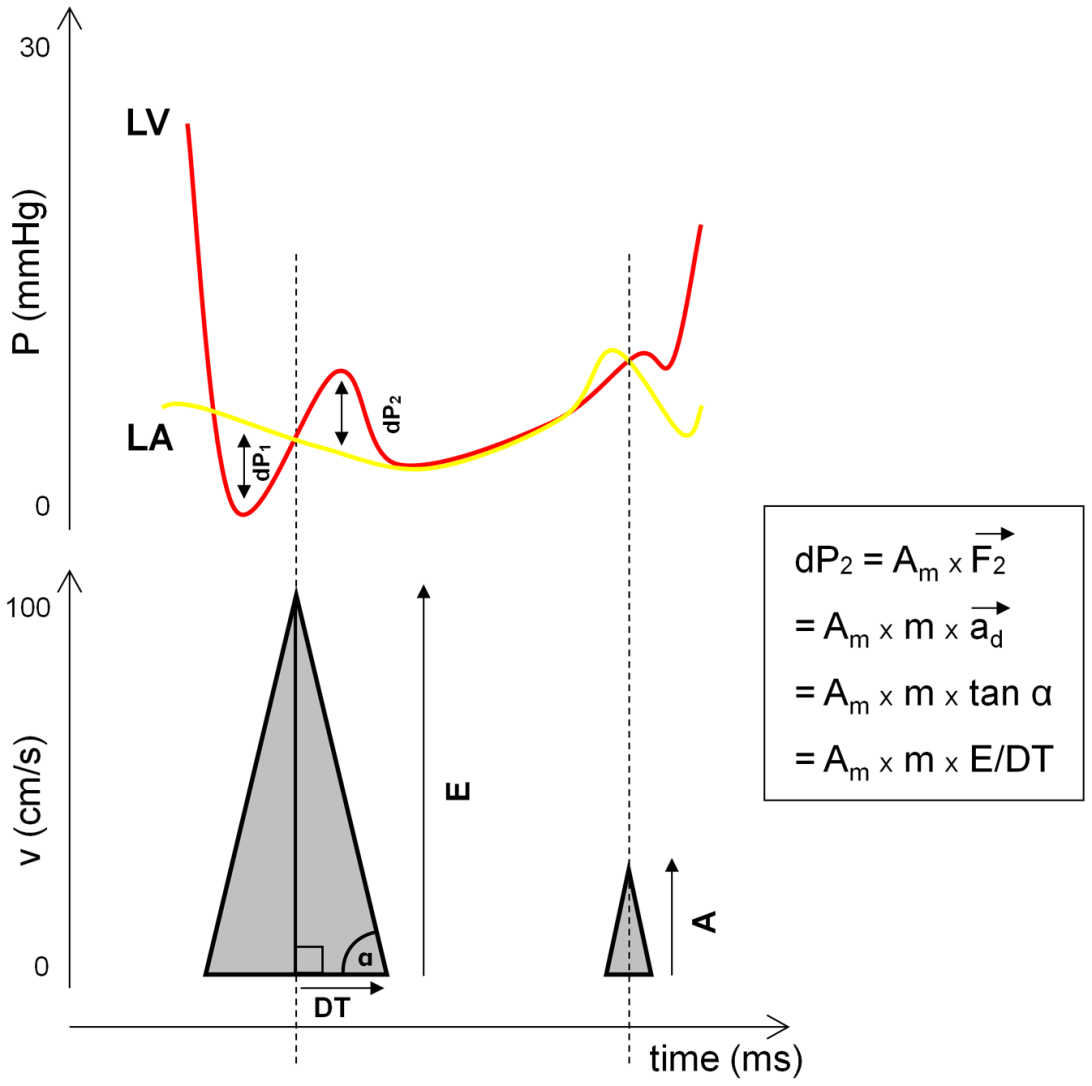
Schematic illustration of the relationship between transmitral Doppler pattern and pressure behaviour in the left atrium and ventricle in diastole. LV: Left ventricle; LA: left atrium; E: peak early diastolic velocity; A: peak late diastolic velocity; DT: deceleration time; dP_1_: atrioventricular pressure gradient; dP_2_: ventriculoatrial pressure gradient; A_m_: area of the mitral orifice; F_2_: force applied to the blood; m: mass of the blood; a_d_: deceleration of early mitral inflow.

**Table 3:** Microscopic parameters of lung remodeling and right ventricular changes in control and pressure overloaded rats at 2, 4 and 6 weeks after aortic banding.

	2 Weeks		4 Weeks		6 Weeks	
	Control (n = 5)	AoB (n = 6)	Control (n = 5)	AoB (n = 7)	Control (n = 5)	AoB (n = 7)
Lung tissue density (%)	44.46±1.87	61.69±7.38	51.88±1.08	81.96±3.19*§	55.17±2.14	74.94±0.97*
Interstitial collagen (%)	4.35±0.32	4.47±0.86	2.89±0.30	3.16±0.68	9.34±1.74	11.03±1.67
Periarteriolar collagen (%)	28.46±1.71	20.07±2.70	22.27±3.44	14.94±1.87	25.04±3.18	21.37±2.63
RVW/BW (g/kg)	0.96±0.06	1.23±0.11	0.84±0.03	1.52±0.11*	0.73±0.01	1.52±0.05*
TAPSE (mm)	1.20±0.24	0.77±0.21	1.04±0.04	0.67±0.07	1.40±0.09	0.76±0.13*
Ts' (cm/s)	1.68±0.09	1.42±0.15	2.28±0.17	1.44±0.12*	2.59±0.10	1.79±0.26*

Data are mean ±SEM.

AoB: aortic banding; RVW/BW: right ventricular weight-to-body weight ratio; TAPSE: tricuspid annular plane systolic excursion; Ts': peak systolic velocity of the lateral tricuspid annulus.

Significantly different vs. age-matched control: *, vs. AoB at 2 weeks: §.

Lung remodeling may result in pulmonary hypertension and subsequently RV disease. To assess if E/DT may also predict RV changes, we measure the right ventricular weight-to-body weight ratio (RVW/BW) and systolic parameters of the RV. As presented in Table 3, RVW/BW as well as TAPSE and Ts' showed similar changing patterns as LW/BW, which indicates sensitive responses of the RV (hypertrophy and systolic dysfunction) to lung remodeling. In agreement with the ability of E/DT to characterize lung remodeling, we found that E/DT correlated well with these parameters, especially with the hypertrophy index RVW/BW (Fig. 8B, 8C, 8D).

## Discussion

The present study addressed for the first time the reliability of echocardiography in evaluating heart failure-induced lung remodeling. By using a well-established model of pressure overload, we found that the novel parameter E/DT best detected and monitored the development of lung remodeling in HFpEF. Following E/DT, the conventional mitral inflow parameters E/A and DT also presented satisfying reliability. In contrast, the tissue Doppler-derived index E/e' correlated only modestly with LW/BW and had lower diagnostic potential compared to mitral inflow parameters in predicting lung remodeling.

The tissue Doppler index E/e' has been suggested as a powerful parameter of diastolic function which can even be used to estimate pulmonary capillary wedge pressure (PCWP) [10], [17], [18]. Based on these findings, the use of E/e' is also recommended in the latest guidelines to diagnose secondary pulmonary disease in HFpEF [8], [9]. Therefore, our results are relevant by showing a relatively low diagnostic accuracy for E/e' in predicting heart failure-induced lung remodeling.

While our findings might be surprising, they are consistent with some recent reports in patients questioning the reliability of E/e' in estimating LV filling pressure. For example, Mullens et al. found no association between E/e' and PCWP in a large population of patients with advanced systolic heart failure [11]. Additionaly, Dokainish et al. showed in a retrospective study a weaker correlation of E/e' with LV diastolic pressure compared to those of E/A and DT [12]. Of note, the patients in these two studies presented with markedly reduced ejection fraction and the results of Mullens et al. have been hotly debated due to some possible limitations [19], [20]. Nonetheless, these results and ours call for critical application of E/e' in evaluating diastolic function and the associated lung remodeling.

It is worth noting that our findings do not disprove the role of the E/e' index as an important diastolic parameter. Clearly, E/e' may be useful in certain contexts. For example, as shown in Fig. 4D and 5D, the early increase in E/e' at 2 weeks may indicate impairments in diastolic function, suggesting that E/e' may help recognize early pathological changes of the LV in pressure overload. However, this early change does not imply a generally high sensitivity of E/e'. In contrast, its sensitivity for the detection of lung remodeling was relatively low as shown in Fig. 7A and 7B. Because the time course of E/e' differs from that of LW/BW, we postulate that other impairments which occur later (e.g. ventricular stiffening), but not those detected by E/e', directly affect overall diastolic function and lung remodeling.

Our study also investigated for the first time the role of the deceleration rate E/DT in assessing heart failure-related lung remodeling. By using a homogeneous animal model, we were able to compare among the echo parameters and found that E/DT is most reliable in detecting and monitoring lung remodeling in pressure overload-induced HFpEF. Furthermore, we also found that E/DT correlated well with RV changes, in particular the RV hypertrophy index. Thus, E/DT may also be useful in predicting RV involvement in HFpEF.

We intensively searched for clinical data on E/DT and found only two studies measuring this parameter. Pozzoli et al. reported a strong correlation of E/DT with PCWP in heart failure patients with or without mitral regurgitation [21]. In a large population with high prevalence of hypertension and diabetes, Mishra et al. found that normalization of DT for E, which is E/DT, predicted cardiovascular events while E or DT alone did not [22]. Despite these findings, E/DT is not mentioned in the latest guidelines [10] and may be unknown to cardiologists. Therefore, our results are relevant by uncovering the diagnostic potential of E/DT in heart failure-induced secondary lung disease. Together with prior data on E/DT, they also suggest advanced validation of this simple and useful, yet overlooked parameter in humans.

In our pressure overloaded rats, we did not observe the flow patterns of the impaired-relaxation or the pseudonormal stage. The E/A ratio was always greater than 1 and DT was decreased at all three time points of assessment. We assume that these two intermediate stages might be present at some time point earlier than 2 weeks, provided that this clinical classification also applies to rats with pressure overload.

Although we showed an excellent diagnostic potential of E/DT in pressure overload, further studies are required to test its role in other models of heart failure. For the clinical use of E/DT, caution should be taken in patients with impaired-relaxation pattern. In this stage, active relaxation of the ventricle is attenuated resulting in reduced atrioventricular pressure gradient (dP_1_ in Fig. 9) and thus in decreased early inflow velocity. The slow ventricular filling may result in a small ventriculoatrial pressure gradient (dP_2_ in Fig. 9) and consequently a decrease in E/DT. Thus, an unusually small E/DT value indicates impaired ventricular relaxation rather than better-than-normal diastolic function.

Because we used the LW/BW ratio to validate diastolic parameters, no conclusions can be made about the reliability of these parameters in estimating ventricular filling pressure. The use of a necropsy marker in spite of intracardiac pressure measurements differs from most studies in this field. However, it is important to note that we focused on the characterization of lung remodeling while previous studies have aimed at estimating ventricular filling pressure. For this purpose, the use of the LW/BW ratio is an advantage because it serves as a direct long-term marker for chronic pulmonary congestion. In contrast, pressure measurements would rather be an indirect approach. From another aspect, they would provide snapshot values, which may be influenced by short-term changes in loading conditions or by anesthetics.

## Conclusions

In pressure overload-induced HFpEF, the mitral Doppler parameter E/DT reliably predicts the presence of lung remodeling and can also be used to monitor its progression. In contrast, the tissue-Doppler index E/e' which has been considered a powerful measure of diastolic function showed a low diagnostic reliability. The results are relevant for the detection and characterization of secondary lung disease associated with heart failure. In addition, they suggest more critical use of E/e' and call for advanced validation of the new and simple parameter E/DT in other models of heart failure and in humans.

## Supporting Information

Figure S1 **Left ventricular myocardial fibrosis.** Masson's trichrome stain of the left ventricle at 6 weeks after AoB reveals marked myocardial fibrosis, which may result in ventricular stiffening and therefore contribute to diastolic dysfunction. Scale bar: 100 µm.
(TIF)

Figure S2 **Macroscopic appearance of lung remodeling.** Lung of a control or pressure overloaded rat at 6 weeks. Lung remodeling involves an increase in lung size and weight. Scale bar: 1 cm.
(TIF)

Figure S3 **Increased tissue density in the remodeled lung.** Masson's trichrome stain. Scale bar: 100 µm.
(TIF)

Figure S4 **Lung remodeling is characterized by cellular infiltration.** Masson's trichrome stain shows massive cellular infiltration without increased lung fibrosis at 6 weeks. Scale bar: 50 µm.
(TIF)

Appendix S1 The rationale why E/DT may characterize pressure overload-induced lung remodeling.
(DOC)
