# Supplementary figures and images for: The E-Wave Deceleration Rate E/DT Outperforms the Tissue Doppler-Derived Index E/e' in Characterizing Lung Remodeling in Heart Failure with Preserved Ejection Fraction
Source: PLoS One. 2013 Dec 3;8(12):e82077. doi: 10.1371/journal.pone.0082077 (PMC3849461; doi:10.1371/journal.pone.0082077)

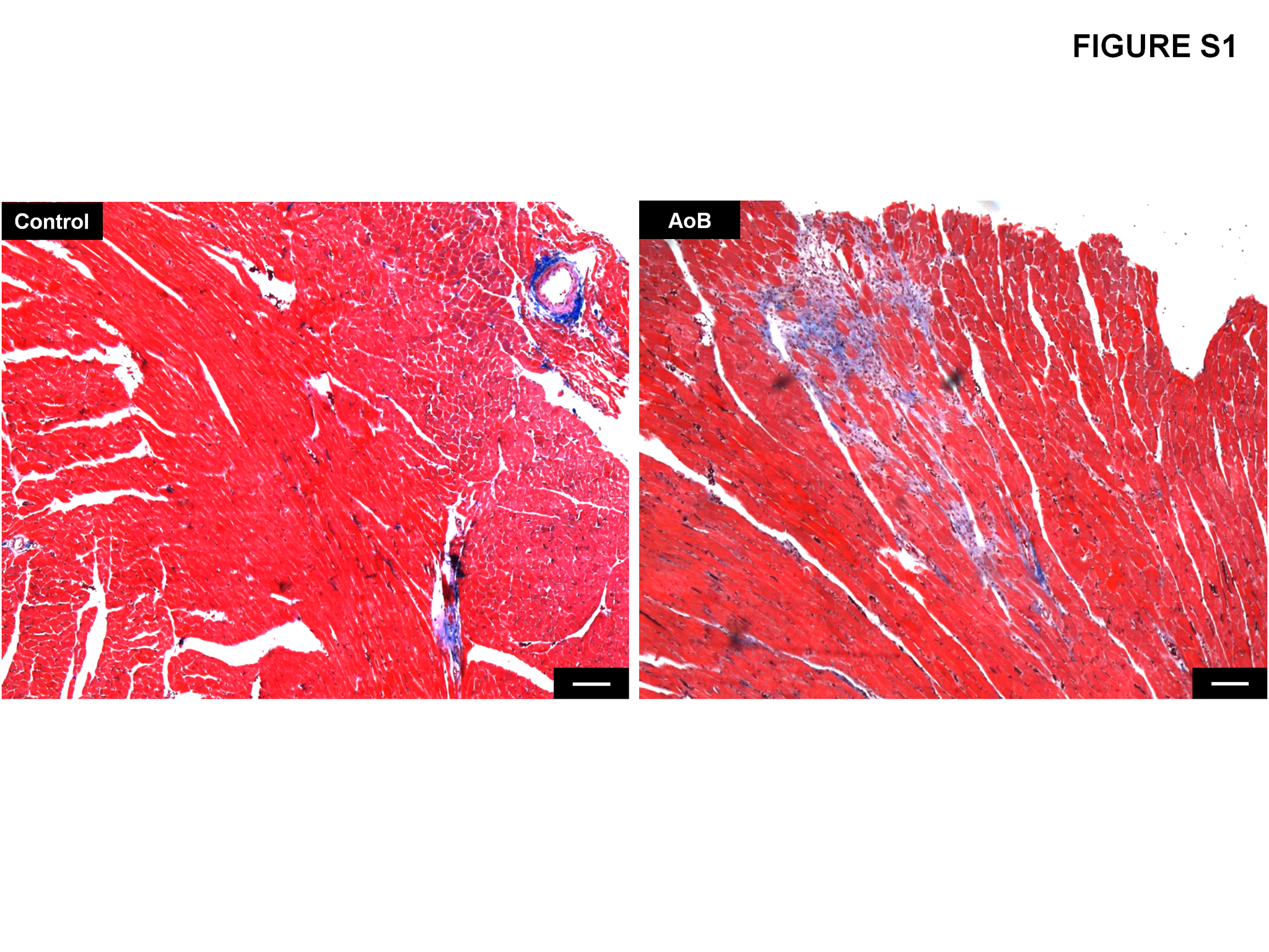

Supplement: Figure S1 — Left ventricular myocardial fibrosis. Masson's trichrome stain of the left ventricle at 6 weeks after AoB reveals marked myocardial fibrosis, which may result in ventricular stiffening and therefore contribute to diastolic dysfunction. Scale bar: 100 µm. (TIF) [file pone.0082077.s001.tif]

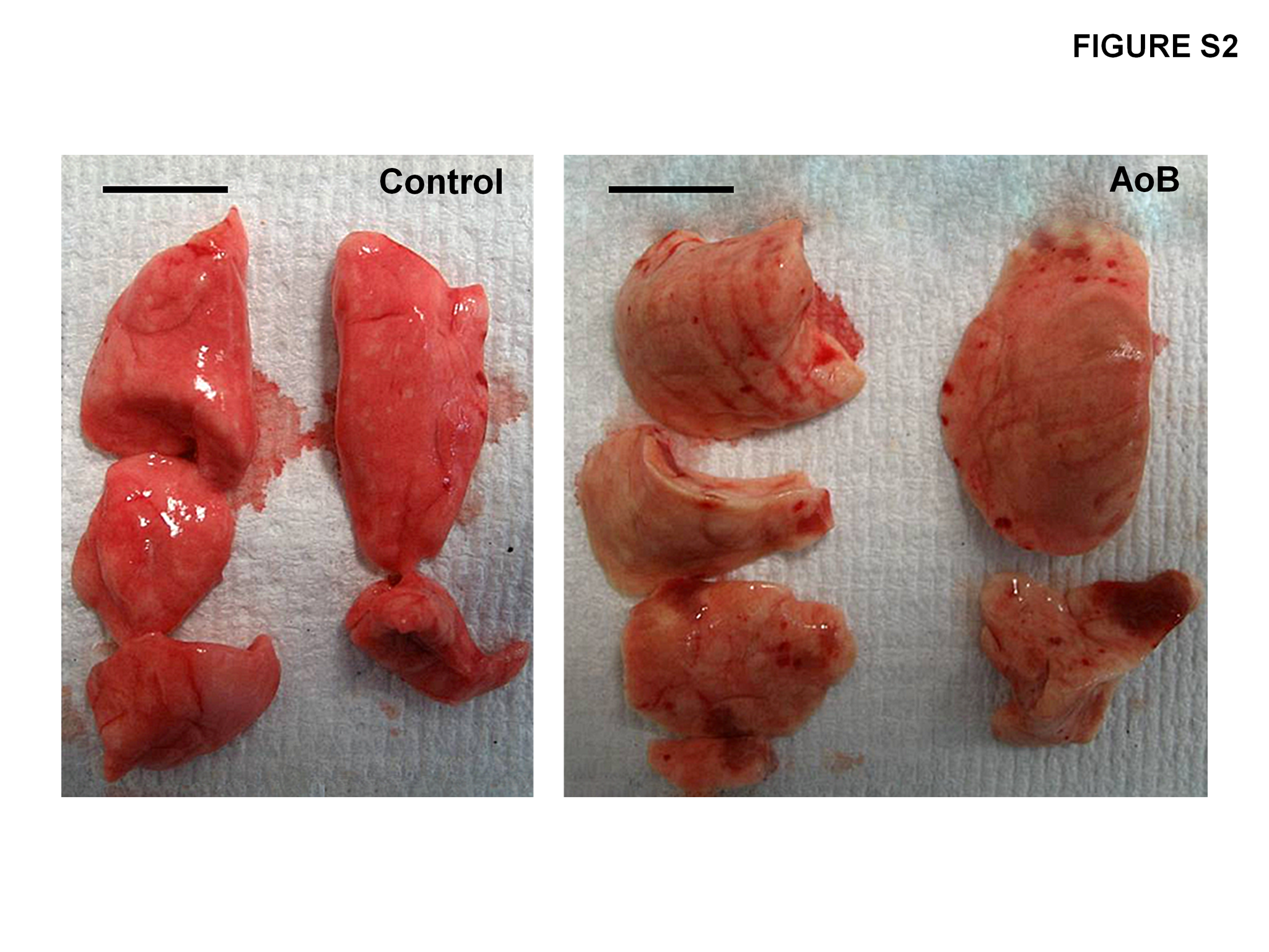

Supplement: Figure S2 — Macroscopic appearance of lung remodeling. Lung of a control or pressure overloaded rat at 6 weeks. Lung remodeling involves an increase in lung size and weight. Scale bar: 1 cm. (TIF) [file pone.0082077.s002.tif]

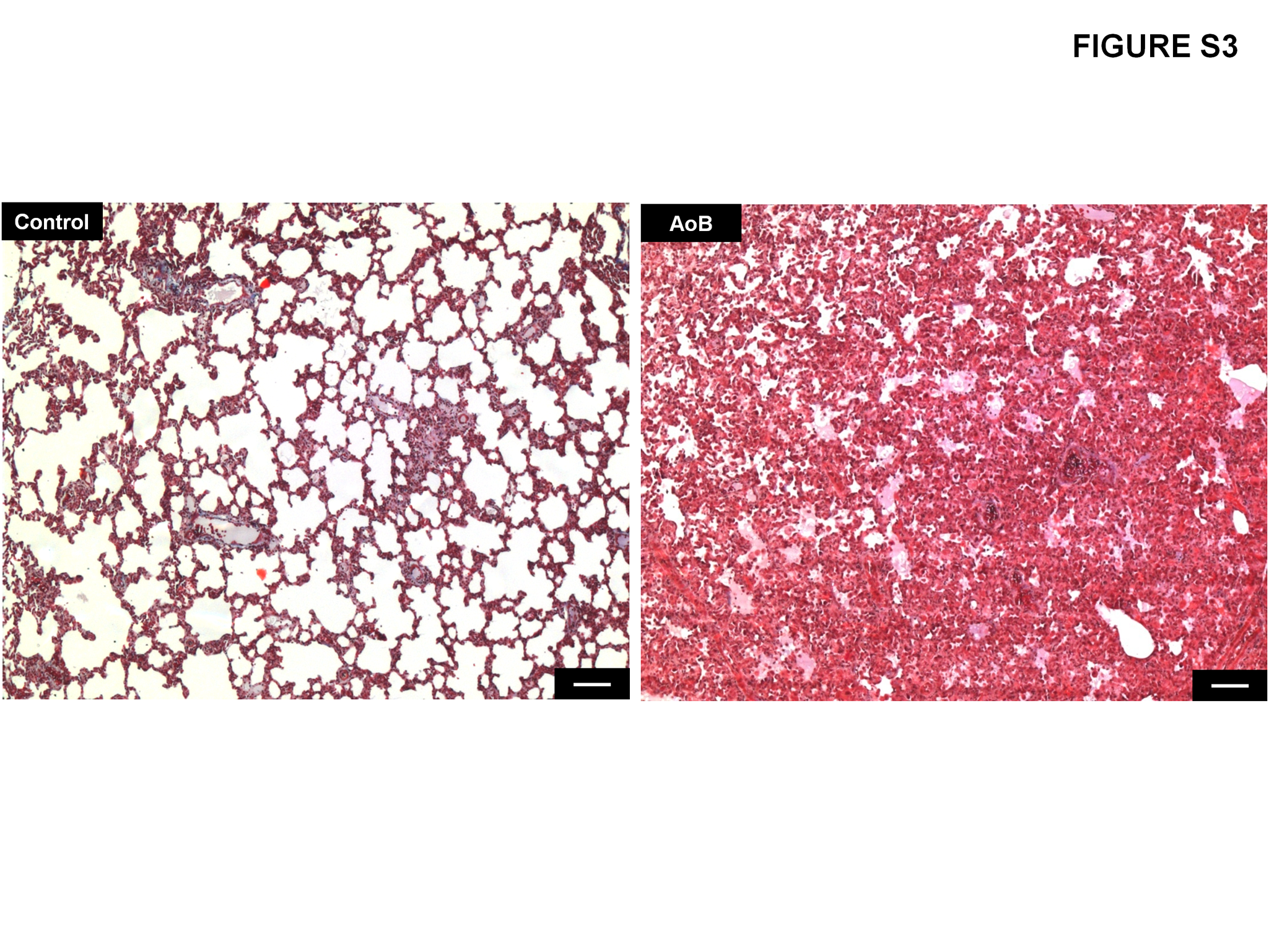

Supplement: Figure S3 — Increased tissue density in the remodeled lung. Masson's trichrome stain. Scale bar: 100 µm. (TIF) [file pone.0082077.s003.tif]

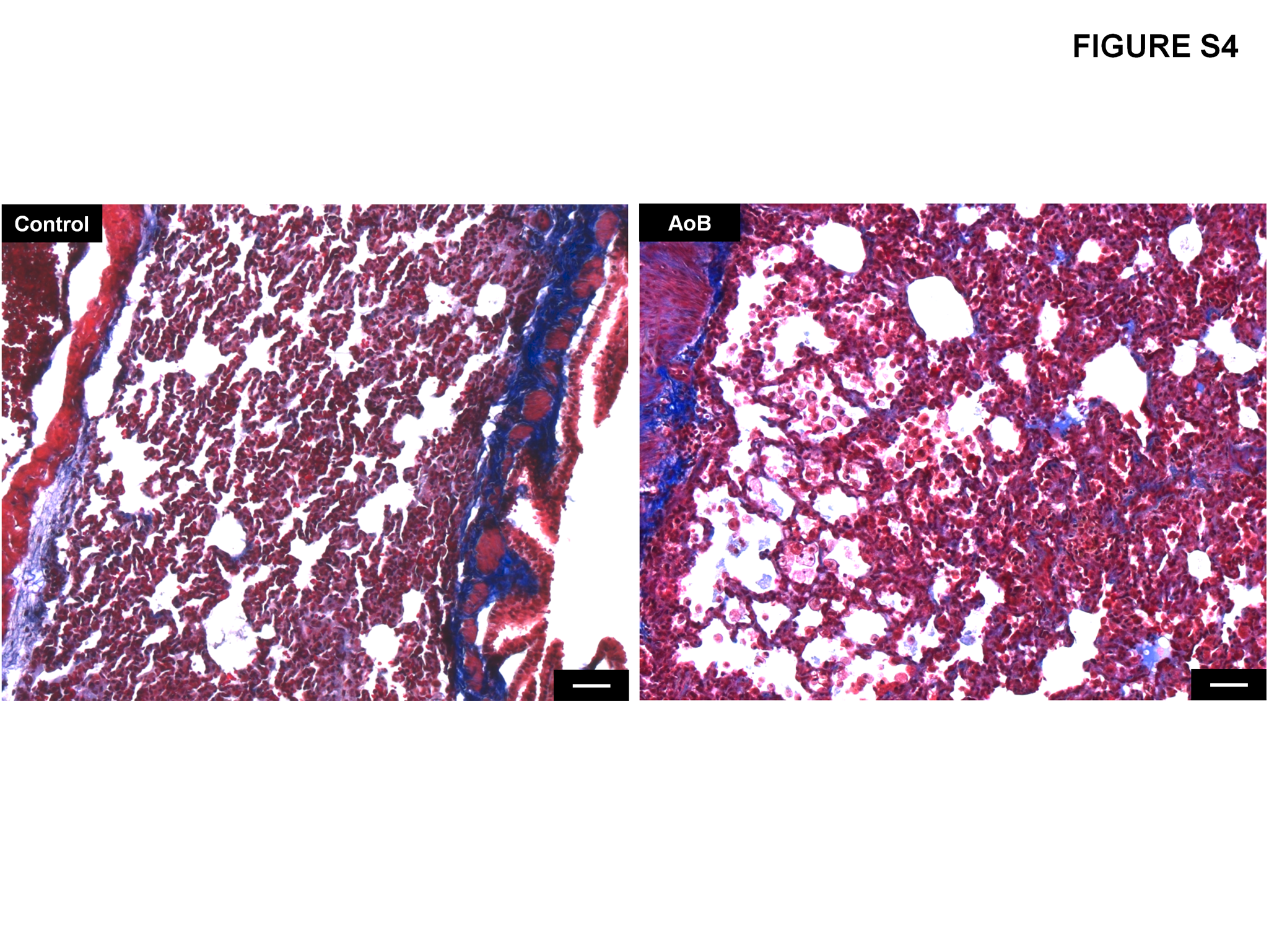

Supplement: Figure S4 — Lung remodeling is characterized by cellular infiltration. Masson's trichrome stain shows massive cellular infiltration without increased lung fibrosis at 6 weeks. Scale bar: 50 µm. (TIF) [file pone.0082077.s004.tif]
