# Supplementary material for: The E-Wave Deceleration Rate E/DT Outperforms the Tissue Doppler-Derived Index E/e' in Characterizing Lung Remodeling in Heart Failure with Preserved Ejection Fraction
Source: PLoS One. 2013 Dec 3;8(12):e82077. doi: 10.1371/journal.pone.0082077 (PMC3849461; doi:10.1371/journal.pone.0082077)
Supplement: Appendix S1 — The rationale why E/DT may characterize pressure overload-induced lung remodeling. (DOC) [file pone.0082077.s005.doc]

**APPENDIX**

**The rationale why E/DT may characterize pressure overload-induced lung remodeling**

Figure 9 illustrates the relationship between hemodynamic changes in left atrium and left ventricle and Doppler pattern of mitral inflow during diastole. (Based on review by Little and Oh) .

At the end of isovolumic relaxation, left ventricular pressure (LVP) becomes lower than left atrial pressure (LAP). The first pressure crossover corresponds to mitral valve opening. The pressure gradient dP_1_ produces the force F_1_ applied to the blood which accelerates the mitral inflow to its peak velocity. The maximal velocity of early diastolic filling (E) is reached exactly at the time of the second pressure crossover. As LVP exceeds LAP, the pressure gradient dP_2_ produces the force F_2_ decelerating the velocity of mitral inflow.

Since pressure is direct proportional to force, one has: (where A_m_ is the area of the mitral orifice)

By Newton’s second law, F_2_ is direct proportional to the deceleration of early diastolic mitral inflow (a_d_), so that: (where m is the mass of the blood)

Although dP_2_ and thus F_2_ change during time, the morphology of early mitral inflow reveals that the deceleration a_d_ is nearly constant, and practically, the steepest slope is measured. Therefore, one has: *a_d_ = tan α = E/DT* (DT is the time for E to be decelerated to zero).

Taken together, dP_2_ is direct proportional to F_2_, to the deceleration a_d_, and thus to E/DT. Since an increase in dP_2_ (e.g. in diastolic dysfunction) may result in pulmonary congestion, we hypothesized that E/DT may detect lung remodeling in HFpEF.
